# Impact of PCSK9 on CTRP9-Induced Metabolic Effects in Adult Rat Cardiomyocytes

**DOI:** 10.3389/fphys.2021.593862

**Published:** 2021-02-11

**Authors:** Susanne Rohrbach, Ling Li, Tatyana Novoyatleva, Bernd Niemann, Fabienne Knapp, Nicole Molenda, Rainer Schulz

**Affiliations:** ^1^Institute of Physiology, Justus Liebig University Giessen, Giessen, Germany; ^2^Excellence Cluster Cardio Pulmonary Institute (CPI), Universities of Giessen and Marburg Lung Center (UGMLC), Member of the German Center for Lung Research (DZL), Justus Liebig University Giessen, Giessen, Germany; ^3^Department of Cardiac and Vascular Surgery, Justus Liebig University Giessen, Giessen, Germany

**Keywords:** PCSK9, adiponectin, metabolism, mitochondria, LRP1, cardiomyocyte

## Abstract

The adipocytokine adiponectin and its structural homologs, the C1q/TNF-related proteins (CTRPs), increase insulin sensitivity, fatty acid oxidation and mitochondrial biogenesis. Adiponectin- and CTRP-induced signal transduction has been described to involve the adiponectin receptors and a number of co-receptors including the Low density lipoprotein receptor-related protein 1 (LRP1). LRP1 is another target of the proprotein convertase subtilisin/kexin-9 (PCSK9) in addition to the LDL-receptor (LDL-R). Here, we investigated the influence of PCSK9 on the metabolic effects of CTRP9, the CTRP with the highest homology to adiponectin. Knockdown of LRP1 in H9C2 cardiomyoblasts blunts the effects of CTRP9 on signal transduction and mitochondrial biogenesis, suggesting its involvement in CTRP9-induced cellular effects. Treatment of adult rat cardiomyocytes with recombinant PCSK9 but not knockdown of endogenous PCSK9 by siRNA results in a strong reduction in LRP1 protein expression and subsequently reduces the mitochondrial biogenic effect of CTRP9. PCSK9 treatment (24 h) blunts the effects of CTRP9-induced signaling cascade activation (AMP-dependent protein kinase, protein kinase B). In addition, the stimulating effects of CTRP9 on cardiomyocyte mitochondrial biogenesis and glucose metabolism (GLUT-4 translocation, glucose uptake) are largely blunted. Basal fatty acid (FA) uptake is strongly reduced by exogenous PCSK9, although protein expression of the PCSK9 target CD36, the key regulator of FA transport in cardiomyocytes, is not altered. In addition, only minor effects of PCSK9 were observed on CTRP9-induced FA uptake or the expression of genes involved in FA metabolism or uptake. Finally, this CTRP9-induced increase in CD36 expression occurs independent from LRP1 and LDL-R. In conclusion, PCSK9 treatment influences LRP1-mediated signaling pathways in cardiomyocytes. Thus, therapeutic PCSK9 inhibition may provide an additional benefit through stimulation of glucose metabolism and mitochondrial biogenesis in addition to the known lipid-lowering effects. This could be an important beneficial side effect in situations with impaired mitochondrial function and reduced metabolic flexibility thereby influencing cardiac function.

## Introduction

Adipose tissue is an important endocrine organ, secreting various adipose-derived cytokines, so called adipokines, which regulate metabolic and non-metabolic functions in our body. Among these adipokines, acrp30 (adiponectin) was shown to increase glucose uptake, insulin sensitivity, fatty acid (FA) uptake and oxidation as well as mitochondrial biogenesis in cardiomyocytes. In addition, adiponectin inhibits myocardial hypertrophy, fibrosis, oxidative stress, and inflammation (Fang and Judd, 2018; Park and Sweeney, 2013). A family of structural and functional adiponectin paralogs, comprising 15 members so far, was discovered and designated as C1q/tumor necrosis factor-alpha-related proteins (CTRPs) some years ago (Wong et al., 2004). The CTRP proteins and adiponectin share a common structure consisting of a signal peptide at the N-terminus, a short variable region, a collagenous domain, and a C-terminal globular domain that is homologous to complement component 1q (Wong et al., 2004). Among these, CTRP9 shares the highest degree of amino acid identity (51%) with the globular domain of adiponectin (Wong et al., 2009) and has been shown to mediate multiple cardioprotective effects (Kambara et al., 2012; Su et al., 2013; Sun et al., 2013; Zhao et al., 2018; Niemann et al., 2020; Zuo et al., 2020). Similar to the effects described for adiponectin, CTRP9 increases glucose uptake and FA oxidation (Wong et al., 2009; Peterson et al., 2013). In addition, CTRP9 activates the peroxisome proliferator-activated receptor-gamma coactivator-1alpha (PGC-1α) and adenosine monophosphate-dependent kinase (AMPK) signaling pathway resulting in increased mitochondrial biogenesis, decreased oxidative stress and slowed aging (Cheng et al., 2016; Li et al., 2018; Niemann et al., 2020). The heart was identified as the organ expressing highest CTRP9 tissue levels, even exceeding adipose tissue where CTRP9 was originally discovered (Wong et al., 2009; Su et al., 2013).

The cellular effects of adiponectin are mediated via the adiponectin receptor AdipoR1 and AdipoR2 and their co-receptor T-cadherin (Yamauchi et al., 2003, 2007; Hug et al., 2004). So far, no specific CTRP receptors have been identified and various studies suggest that the CTRP effects are mediated via AdipoR1 (Zheng et al., 2011; Kambara et al., 2012; Cheng et al., 2016). Interestingly, the loss of AdipoR1 and AdipoR2 does not abolish all adiponectin or CTRP effects, suggesting the existence of additional receptors and co-receptors (Park et al., 2009; Wong et al., 2009; Niemann et al., 2020). Among these is the low-density lipoprotein receptor-related protein 1 (LRP1)/calreticulin co-receptor system (Maruyama et al., 2011; Potere et al., 2019; Niemann et al., 2020).

The proprotein convertase subtilisin/kexin 9 (PCSK9) enhances the degradation of the LDL-R, resulting in increased circulating LDL cholesterol. In addition, PCSK9 is capable of inducing degradation of other membrane receptors including LRP1 and CD36, the main sarcolemmal transporter for long-chain FA (Canuel et al., 2013; Norata et al., 2014; Demers et al., 2015; Filippatos et al., 2018). The current study therefore investigated the questions of whether: (a) LRP1 is involved in CTRP9-induced effects in cardiomyocytes. (b) PCSK9 alters CTRP9-induced signal transduction. (c) PCSK9 influences the CTRP9-induced changes in mitochondrial biogenesis and glucose or fatty acid metabolism.

## Materials and Methods

### Isolation and Treatment of Rat Adult and Neonatal Cardiomyocytes

Adult ventricular cardiomyocytes were isolated from Wistar rats (Janvier) as described in detail previously (Schluter and Schreiber, 2005). Briefly, hearts were excised under deep anesthesia, transferred rapidly to ice-cold saline, and mounted on the cannula of a Langendorff perfusion system. Hearts were perfused first for 10 min in a non-re-circulating manner with a calcium-free perfusion buffer, then for 20–25 min in a re-circulating manner in a buffer supplemented with collagenase and 25 μmol/l calcium. Afterward both ventricles were minced and incubated for another 5 min in re-circulating buffer. The resulting cell solution was filtered through a 200 μm nylon mesh. The suspension was centrifuged at 25 × *g* for 10 min to pellet down the cardiomyocytes, while the supernatant contained mostly the endothelial cells and fibroblasts. Cardiomyocytes were re-suspended in buffer with a stepwise increase in calcium and finally transferred to culture medium (M199 supplemented with 2 mM carnitine, 5 mM creatine, and 5 mM taurine). Adult cardiomyocytes were attached to culture dishes by pre-coating of the dishes with 4% FCS.

Ventricular cardiomyocytes from 2 to 3-day-old Wistar rats were isolated and cultivated as described before (Engel et al., 2003; Novoyatleva et al., 2010). Briefly, hearts were dissected, minced, and trypsinized. Afterward, cardiomyocytes were enriched by pre-plating for 90 min at 37°C. The suspension of non-attached cardiomyocytes was collected, centrifuged for 5 min at 330 × *g*, resuspended in DMEM F12 + GLUTAMAX containing 100 U/mg/ml penicillin/streptomycin, 3 mM sodium pyruvate, 0.5× insulin-transferrin solution, 0.2% bovine serum albumin and 0.1 mM ascorbic acid. Cells were cultured for 48 h in the presence of 20 μM cytosine β-D-arabinofuranoside and 5% horse serum to prevent proliferation of non-myocytes. Animal experiments were approved by the local committee for care and use of laboratory animals (Regierungspräsidium Giessen, Nr. 596_M and Nr. 697_M) and were executed in accordance to the Guide for the Care and Use of Laboratory Animals Directive 2010/63/EU of the European Parliament.

SiRNA (FlexiTube siRNA, Qiagen) oligonucleotides were transfected to neonatal cardiomyocytes at a concentration of 0.5 nmol/l with Lipofectamine^®^ RNAiMAX (Thermo Fisher Scientific). The sequences (Table 1) had been digitally searched and no similarity to other genes was found in current databases. The control cells were transfected with control siRNA oligonucleotides with no known target in mammalian genomes. 48 h after siRNA transfection, cells were treated with the indicated substances in serum-free medium. In adult cardiomyocytes, PCSK9 and control siRNA (FlexiTube siRNA, Qiagen) oligonucleotides were directly supplied to the cells at a concentration of 5 nmol/l without utilization of a transfection reagent as described before (Schluter et al., 2017).

**TABLE 1:** siRNA.

	Sense strand sequence
PCSK9 rat	5′-GGUAUAGCCGGAUCCUUAATT-3′
LRP1 rat	5′-CGUUGGUUAUGCACAUGAATT-3′
PCSK9 mouse	5′-GGUGGAGGUGUAUCUCUUATT-3
LRP1 rat	5′-CCAGAACATCCTAGCTACGTA-3
LDL-R rat	5′-ATGGGCGGACCGACTGTGAAA-3

Cardiomyocytes were treated with exogenous PCSK9 (0.5 μg/ml) for 24 h and afterward stimulated with CTRP9 (4 μg/ml). CTRP9 was expressed in E. coli and isolated and purified as described previously (Niemann et al., 2020). Rat acrp30 (BioCat GmbH) was also utilized at 4 μg/ml which is in accordance with plasma levels in rats *in vivo* (Ye et al., 2004; Boullu-Ciocca et al., 2008).

### H9C2 Cardiomyoblast and AML12 Hepatocyte Culture and siRNA Transfection

The H9C2 rat cardiomyoblast and the AML12 mouse hepatocyte cell line were obtained from the American Type Culture Collection. H9C2 cells were maintained in DMEM medium supplemented with 10% FCS and 1% penicillin/streptomycin under an atmosphere of 5% CO_2_ in air at 37°C. AML12 were maintained in DMEM:F12 medium supplemented with 10% FBS, 5 μg/ml insulin, 5 μg/ml transferrin, 5 ng/ml selenium, and 40 ng/ml dexamethasone under an atmosphere of 5% CO_2_ in air at 37°C. Twenty four hours before transfection, cells were trypsinized and transferred to 6-well plates (5 × 10^5^ cells/well). Transfection was performed as described above for neonatal rat cardiomyocytes.

### RNA Isolation and qPCR

Total RNA from cardiomyocytes, microvascular endothelial cells, cardiac fibroblasts and tissue samples was isolated using TriFast (Peqlab) according to the manufacturer’s instructions. Prior to cDNA synthesis, integrity and quality of the RNA was confirmed by gel electrophoresis and the concentration determined by measuring UV absorption. Reverse transcription of RNA samples (500 ng total RNA) was carried out for 30 min at 42°C using the SuperScript^TM^ III First-Strand cDNA Synthesis Kit (Thermo Fisher Scientific). RT-PCR was performed for PCSK9, ND1, Cox I, LRP1, PGC-1alpha, Tfam, FABP3, CD36, LCAD, MCAD, VLCAD, GLUT-1, GLUT-4, hexokinase, PFK1, beta-globin, HPRT1, GAPDH and 18S rRNA. Amplification products were subjected to electrophoresis through 1.5% agarose gels, stained with GelRed (VWR) and visualized with the Fusion FX7 imaging system (Peqlab). PCR products were excised from the gel, purified and directly sequenced. Real-time PCR and data analysis were performed using the Mx3000P Multiplex Quantitative PCR System (Stratagene) as described previously (Niemann et al., 2011). Each assay was performed in duplicate and validation of the PCR runs was assessed by evaluation of the melting curve of the PCR products (primer sequences in Table 2). Every reaction was performed as duplicates and quantified with the ΔΔ*C*_*T*_-method. Threshold cycles (*C*_*T*_) of target genes were normalized to the mean of the housekeeping genes 18S rRNA, HPRT1, and GAPDH. The resulting Δ*C*_*T*_ values were compared to untreated control cells or young mice receiving control diet and relative mRNA expression was calculated by *R* = 2^–ΔΔCT^.

**TABLE 2:** Primer sequences.

	GenBank accession #	Forward primer	Reverse primer
PCSK9 rat	NM_199253.2	CGG GGC TAT GTC ATC AAG GTT	TGC TCT GGG CGA AGA CTA ATG
ND1 rat	KM577634.1	CCC AAC ATC GTA GGC CCA TA	GAG AGG GTT GGG GCG ATA AT
Cox I rat	KM577634.1	GAT TCT TCG GAC ACC CAG AA	AGG CTC GCG TGT CTA CAT CT
LRP1 rat	NM_001130490.1	TAC CAA CCA GGC CAC AAG AC	ATT CGG ATC ACA GAC GTG GG
PGC-1alpha rat	NM_031347	CCG AGA ATT CAT GGA GCA AT	GTG TGA GGA GGG TCA TCG TT
Tfam rat	NM_031326.1	ATG GGC TTA GAG AAG GAA GCC	GTG ACT CAT CCT TAG CCC CC
FABP3 rat	NM_024162.1	TCA AGT CGG TCG TGA CAC TG	GCC TCC TTC TCG TAA GTC CG
CD36 rat	NM_031561.2	TGC AGG TCA ACA TAC TGG TCA A	CCC GGT CAC TTG GTT TCT GA
LCAD rat	NM_012819.1	TCA TGC AAG AGC TCC CAC AG	GCA GCT GTC CAC AAA AGC TC
VLCAD rat	NM_012891.2	CCT CTG CCC AGC GAC TTT AT	CCT CTG CCC AGC GAC TTT AT
MCAD rat	NM_016986.2	GGT CTT GGC CTG GGA ACT TT	AGT AGG CAC ACA TCA TCG GC
GLUT-1 rat	NM_138827	TGT GTT CTA CTA CTC AAC GAG CA	CAA TGA GAT GCA GGG TCC GA
GLUT-4 rat	NM_012751.1	GGC TGT AGC TGG TTT CTC CA	AAA TGT CCG GCC TCT GGT TT
Hexokinase rat	NM_012735.2	TCG CAT ATG ATC GCC TGC TT	AGC TCC TAG CCC TTT CTC CA
PFK1 rat	NM_031715.1	TGG GGC TGA CAC TTT TCG TT	GTC GAT TGA GCC AAC CAG GA
Beta-globin rat	NM_001113223.1	GCC TGT GGG GAA AGG TGA ATG	CTT CAC CTG GGG GTT ACC CAT
mtDNA rat	KM577634.1	AGT GAA GGG GCG GAC TCA TA	GAG GTC ACC CCA ACC GAA AT
18S rRNA	NR_046237	TGG AGC GAT TTG TCT GGT TA	ACG CCA CTT GTC CCT CTA AG
GAPDH rat	NM_017008.4	CAC CAT CTT CCA GGA GCG AG	GAA GGG GCG GAG ATG ATG AC
HPRT-1 rat	NM_012583.2	ACC AGT CAA CGG GGG ACA TA	ATT TTG GGG CTG TAC TGC TTG A
CD36 mouse	NM_007643.4	GGT CAA CAT ATT GGT CAA GCC A	TCT ACC ATG CCA AGG AGC TT
LDL-R mouse	NM_010700.3	GTG TGA CCG TGA ACA TGA CTG	CCT TGT CCA AGC TGA TGC AC
LRP1 mouse	NM_008512.2	TGT GAA GTG CTC CTG CTA CG	TGA AGG TCA ATG CGC CTG AT
PCSK9 mouse	NM 153565.2	TTA TCC CAG CAT GGC ACC AG	GTG ACC CTG CCC TCA ATC TC
GAPDH mouse	NM_008084.3	CAT CAC CAT CTT CCA GGA GCG	CGT TTG GCT CCA CCC TTC AA
HPRT-1 mouse	NM_013556.2	GAT CAG TCA ACG GGG GAC AT	AGA GGT CCT TTT CAC CAG CAA

### Measurement of mtDNA Content

For mtDNA quantification, DNA was extracted and purified from cardiomyocyte samples with the DNeasy Blood & Tissue Kit (Qiagen). The mtDNA and nuclear DNA copy number was determined by Real-time-PCR of 16S rRNA and beta-globin as described before. The relative fold change was then calculated using the ΔΔ*C*_*T*_ method.

### Protein Isolation and Western Blotting

Cells and tissues were homogenized in a buffer containing 50 mM Tris HCl, 150 mM NaCl, 5 mM EDTA, 0.1% SDS, 1% sodium deoxycholate and protease and phosphatase inhibitor cocktails (Sigma) to isolate whole cell lysates. Isolation of the membrane fraction was performed with the Mem-PER^TM^ Plus Membrane Protein Extraction Kit (Thermo Fisher Scientific). After sonication and centrifugation, protein concentration was measured with the Pierce^TM^ BCA Protein Assay Kit (Thermo Fisher Scientific). 50 μg of protein were loaded on a SDS-PAGE gel and transferred to a nitrocellulose membrane. Following blocking, filters were incubated with antibodies directed against, phospho-AMPK (Thr172), alpha-AMPK, phospho-Akt (Thr308), total-Akt, phospho-AS160 (Thr642), total-AS160 (all Cell Signaling Technology), PCSK9 (Abcam), ND1 (Abcam), Cox I (Thermo Fisher Scientific), Na-K-ATPase and GAPDH (both Abcam). In order to rule out unspecific binding, the PCSK9 antibody was tested in tissue from PCSK9 knockout mice (Supplementary Figure 3C). After incubation with peroxidase-conjugated secondary antibody, blots were subjected to the enhanced chemiluminescent detection method with the Fusion FX7 imaging system (Peqlab).

### Isolation and Analysis of Membrane Fraction

Isolation of the membrane and cytosolic fraction from H9C2 cells and rat cardiomyocytes was performed with the Mem-PER^TM^ Plus Membrane Protein Extraction Kit (Thermo Fisher Scientific). After measurement of the protein concentration with the Pierce^TM^ BCA Protein Assay Kit (Thermo Fisher Scientific) 20 μg of each fraction were loaded on a SDS-PAGE gel and transferred to a nitrocellulose membrane. Following blocking, filters were incubated with antibodies directed against GLUT-4 (from S. W. Cushman), LRP1 (Abcam), LDL-R (Abnova), CD36 (Abcam) as well as Na-K-ATPase (Abcam) as a marker for the membrane fraction and GAPDH (Abcam) as a marker for the cytosolic fraction. After incubation with peroxidase-conjugated secondary antibody, blots were subjected to the enhanced chemiluminescent detection method with the Fusion FX7 imaging system (Peqlab).

### Measurement of Glucose Uptake

Cells were starved in DMEM without serum and glucose for 3 h, then stimulated with CTRPs in serum-free DMEM containing 0.05 μCi/ml 2-[1-^14^C]-2-deoxyglucose (2DG) and 50 μM 2DG for 1 h. Cells were then washed three times with ice-cold PBS, treated with 500 mM NaOH and incubated overnight at 37°C. Radioactivity was measured by liquid scintillation counting. Glucose uptake was expressed as the fold-increase over the basal level and calculated in relation to the protein concentration of the sample.

### Measurement of Fatty Acid Uptake

The use of fluorescently labeled FA to study their cellular uptake was adapted from adipocytes and has been used in cardiomyocytes before (Wu et al., 2020). Adult cardiomyocytes were plated into 24-wells. For detecting FA uptake, 10 μM of the fluorescent long-chain FA analog BODIPY^TM^500/510 C1, C12 (Thermo Fisher Scientific) was loaded into isolated cardiomyocytes for 5 min and the cells were then washed with 0.5% BSA in PBS twice. Intracellular fluorescence was measured immediately using a Fluostar Optima microplate fluorometer (BMG Laboratories; excitation 488 nm, emission 515 nm, cut-off 495 nm). The cells were then lysed in a buffer containing 50 mM Tris HCl, 150 mM NaCl, 5 mM EDTA, 0.1% SDS, 1% sodium deoxycholate and protease and phosphatase inhibitor cocktails to measure protein concentration of the samples.

### Measurement of Mitochondrial Enzyme Activities

10^7^ adult rat cardiomyocytes were homogenized in a solution containing 50 mM of Tris buffer (pH 7.5), 100 mM potassium chloride, 5 mM MgCl_2_, and 1 mM EDTA using a glass/glass homogenizer. Assays were run in duplicates with two different quantities of sample. Enzyme activities were referenced to the activity of mitochondrial marker enzyme citrate synthase (CS) as measured in the same sample. The activity of complex I, complex II and complex IV were performed as described previously (Niemann et al., 2011) using a Cary 50 spectrophotometer (Varian Instruments, Australia).

### Construction of Adenoviral Plasmids Expressing PCSK9 and Adenovirus Production

The PCSK9 sequence used in this study for mouse wild-type PCSK9 or constitutive active mouse PCSK9 was obtained by RT-PCR from mouse liver or pAAV/D377Y-mPCSK9 (Addgene #58376) respectively. After cloning into pENTR^TM^/D-TOPO^®^ and recombination into the vector pAd/CMV/V5 (both Thermo Fisher Scientific), 293A producer cells containing a stably integrated copy of the adenoviral E1 gene, were transfected with the adenoviral plasmids in order to prepare the crude adenoviral lysate. Amplification of the adenovirus was performed by infecting 293A producer cells with this crude lysate. An adenoviral plasmid with LacZ was generated as a control (pAd/CMV/V5 LacZ). After determination of the titer of the adenoviral stocks, the viral supernatant was added to HepG2 cells. The supernatants of HepG2 cells containing wild-type mouse PCSK9, a gain-of-function (GOF) variant of mouse PCSK9 (D377Y) or LacZ in Opti-MEM were collected 48 h after infection and utilized to treat rat cardiomyocytes after measuring the PCSK9 content.

### ELISA

The PCSK9 concentrations in the supernatant of HepG2 cells and in the serum of mice were measured by using a commercial Enzyme-Linked-Immunosorbent-Assay (Boster Biological Technology) according to the manufacturers’ instructions.

### Cell-Based Assay for PCSK9 Activity

In order to test the activity of PCSK9 isolated from the supernatant of HepG2 cells, we measured diI-LDL uptake in a cell-based assay as described previously by others (Benjannet et al., 2010). For this purpose, H9C2 cells were seeded in a 96-well (20.000/well). On the next day the medium was changed to DMEM containing 10% lipoprotein-deficient serum and 100 nM simvastatin for 16 h. The cells were then pre-incubated for 2 h in the presence of PCSK9 or LacZ (control), followed by the addition of 5 μg/ml fluorescently labeled LDL (diI-LDL) for another 2 h. The uptake of diI-LDL was stopped by the addition of 4% formaldehyde with 10 μM Hoechst 33342 for 20 min. Cells were washed twice with PBS and DNA content (Hoechst 33342) was measured at excitation/emission 360/460 nm using a Fluostar Optima microplate fluorometer (BMG Laboratories). Afterward the cells were lysed in 0.1 N NaOH, 0.1% SDS, followed by fluorescence reading for diI-LDL at excitation/emission at 520/580 nm. For data analysis, the fluorescence ratio of diI-LDL/Hoechst 33342 was utilized.

### Animals and Diet Protocol

Male young and old C57Bl/6 mice (3 and 18 months at the beginning of the study, respectively, *n* = 14 per group) were obtained from Janvier (Germany), caged individually and received either control diet (diet 1340; 2400 cal/g, Altromin), LFD (D12450B, 3,850 cal/g, Research Diets) or HFD (D12451; 4,730 cal/g, Research Diets) for 16 weeks as described in detail previously (Aurich et al., 2013). The control diet provided 10 kcal% fat, 71 kcal% carbohydrate, and 19 kcal% protein, the LFD provided 10 kcal% fat, 70 kcal% carbohydrate, and 20 kcal% protein and the HFD provided 45 kcal% fat (mainly from lard), 35 kcal% carbohydrates, and 20 kcal% proteins.

### Statistical Analysis

All data are presented as mean ± SEM. Statistical analyses were performed with SigmaStat 3.5 software (Systat Software, Inc.). Data were analyzed for normal distribution (Shapiro–Wilk test) and variance (Levene test) and subsequently analyzed using student *t-*test or ANOVA with *post hoc* analysis as appropriate. *P*-values of <0.05 were considered statistically significant.

## Results

### Role of LRP1 and PCSK9 in Mediating the CTRP9 Effects

**FIGURE 1:**
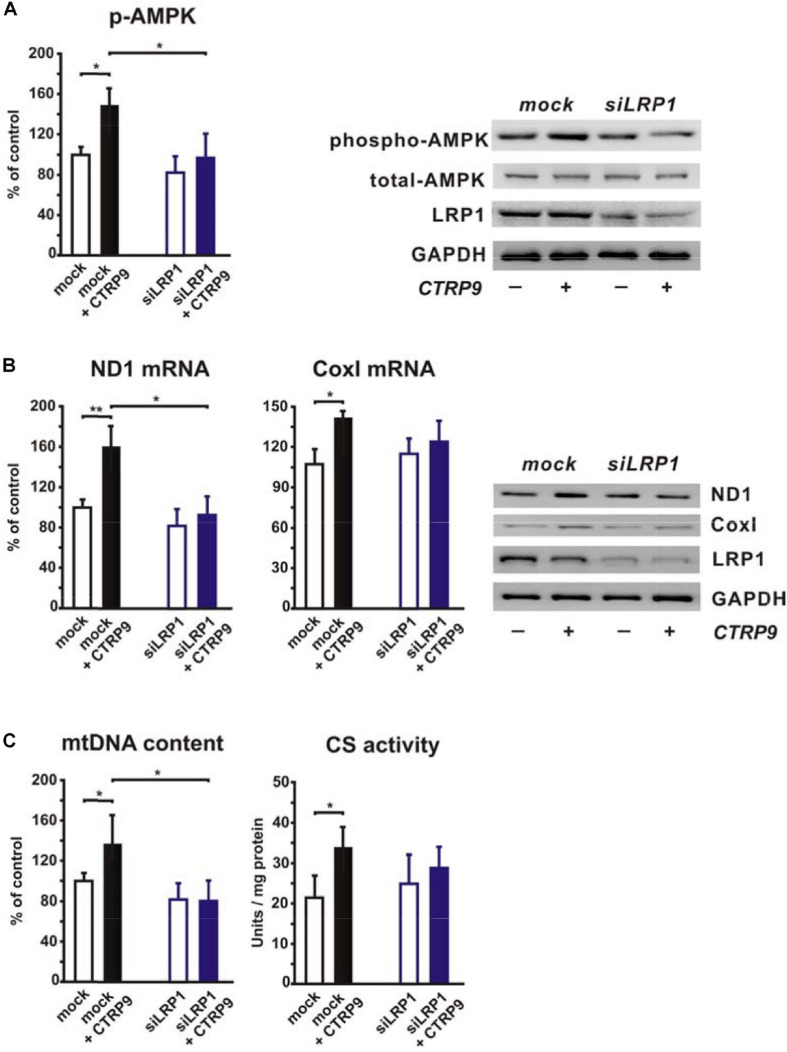
Impact of LRP1 on CTRP9-induced mitochondrial biogenesis. H9C2 cardiomyoblasts were transfected with siRNA directed against rat LRP1 or mock siRNA for 48 h and subsequently treated with CTRP9 (4 μg/ml) for the indicated times. **(A)** Cells were treated with CTRP9 (4 μg/ml) for 20 min and afterward analyzed by Western Blotting for p-AMPK, total-AMPK and LRP1. Representative Western blots and densitometry of protein data are shown. GAPDH served as a loading control. **(B)** Cells were treated with CTRP9 (4 μg/ml) for 24 h. mRNA expression of ND1 and Cox I as determined by real-time PCR (left panels) and representative Western blots for ND1, Cox I, LRP1, and GAPDH (right panel) are shown. **(C)** Cells were treated with CTRP9 (4 μg/ml) for 24 h. Genomic DNA was isolated from the cells and afterward analyzed by Real-time PCR for the expression of a mitochondrial transcript and a nuclear transcript in order to estimate mtDNA content (left panel). Citrate synthase (CS) activity (right panel) was measured in H9C2 cell lysates. All values were normalized to total protein content of the respective sample. All data are mean ± SEM, 4 independent experiments, *n* = 8 per group, *p < 0.05, **p < 0.01.

**FIGURE 2:**
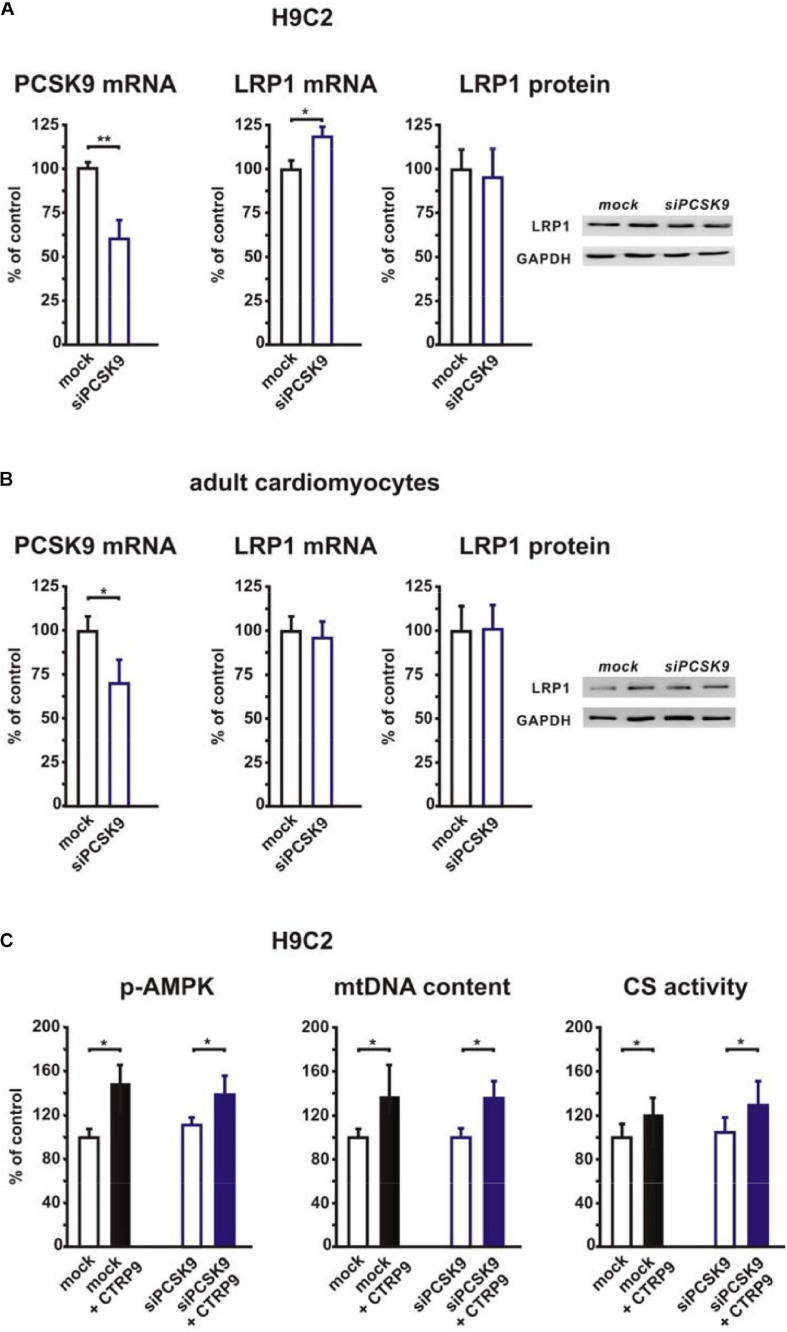
Impact of PCSK9 knockdown on LRP1 expression and CTRP9-induced mitochondrial biogenesis. **(A)** H9C2 cardiomyoblasts were transfected with siRNA directed against rat PCSK9 or mock siRNA for 48 h. Afterward PCSK9 mRNA, LRP1 mRNA, and LRP1 protein expression were analyzed. mRNA expression of PCSK9 and LRP1 as determined by real-time PCR and densitometry of protein data and representative Western blots for LRP1 and GAPDH are shown. **(B)** Adult rat cardiomyocytes were incubated with siRNA directed against rat PCSK9 or mock siRNA for 48 h and afterward analyzed for PCSK9 mRNA and LRP1 mRNA or protein expression by real-time PCR or Western blotting. GAPDH served as a loading control. **(C)** H9C2 cardiomyoblasts were transfected with siRNA directed against rat PCSK9 or mock siRNA for 48 h and thereafter treated with CTRP9 (4 μg/ml) for the indicated times. Activation of AMPK was analyzed after 20 min stimulation with CTRP9 and mtDNA content and citrate synthase activity after 24 h stimulation with CTRP9 as described in Figure 1. All data are mean ± SEM, 4 independent experiments, *n* = 8–16 per group, *p < 0.05; **p < 0.01.

In order to elucidate the potential role of the co-receptor LRP1, we stimulated LRP1 siRNA-treated H9C2 cardiomyoblasts with CTRP9 and analyzed changes in signal transduction as well as in markers of mitochondrial biogenesis. The CTRP9-induced activation of AMPK, which has been shown to be involved in mediating the CTRP9 effects on mitochondria (Cheng et al., 2016; Li et al., 2018; Niemann et al., 2020), was significantly blunted following knockdown of LRP1 with siRNA (Figure 1A). Similarly, various indicators of mitochondrial biogenesis such as increased mRNA and protein expression of ND1, part of complex I of the respiratory chain, or Cox I, part of complex IV of the respiratory chain, were significantly blunted in H9C2 cells with LRP1 knockdown (Figure 1B). Accordingly, mtDNA content and citrate synthase (CS) activity were increased in mock transfected, CTRP9-treated cells H9C2 cells but not after LRP1 knockdown (Figure 1C), suggesting that LRP1 is indeed involved in mediating these CTRP9 effects. Next we investigated the impact of endogenous PCSK9 on the LRP1 expression in H9C2 cardiomyoblasts and adult rat cardiomyocytes. Knockdown of PCSK9 (Figures 2A,B) did not alter the mRNA or protein expression of LRP1 in H9C2 cardiomyoblasts (Figure 2A) or adult cardiomyocytes (Figure 2B). Similarly, PCSK9 knockdown in cardiomyoblasts did not alter the CTRP9-induced increase in AMPK activation, mtDNA content or CS activity (Figure 2C). However, when we utilized the hepatocyte cell line AML12, an increase in LRP1 protein expression was observed following PCSK9 knockdown (Supplementary Figure 1). Unlike LRP1 protein, which was highly expressed in left ventricular (LV) tissue and in adult rat cardiomyocytes (Supplementary Figures 2A,B), PCSK9 protein expression was low in the LV (Supplementary Figure 2C). Similarly, PCSK9 mRNA expression was significantly higher in various rat organs including small intestine, ovary, liver, adipose tissue and kidney than in the LV (Supplementary Figure 2D). Interestingly, there was also a considerable PCSK9 mRNA and protein expression in visceral adipose tissue, although the expression levels of liver were not reached (Supplementary Figures 2C–E). Compared to adult cardiomyocytes, liver PCSK9 mRNA expression was approximately 5.000 times higher (not shown). Accordingly, PCSK9 protein expression was barely detectable in unstimulated cardiomyocytes even after loading high amounts of protein lysate (Supplementary Figure 3A). PCSK9 mRNA expression was similarly low in H9C2 cells but more than 300-fold higher in neonatal rat cardiomyocytes (Supplementary Figure 3B).

### PCSK9 Release *in vivo*

**FIGURE 3:**
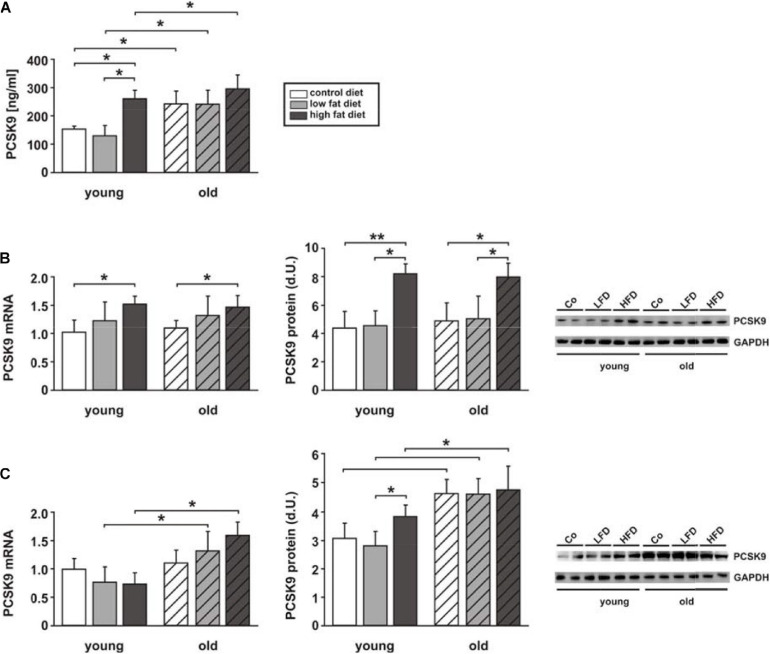
Impact of diet-induced obesity on PCSK9 release and expression. Measurements were performed in samples of young and old mice receiving control diet, low fat diet (LFD) or high fat diet (HFD) for 16 weeks. **(A)** Plasma PCSK9 was measured by ELISA at the end of the study. **(B)** Results from qPCR analyses on PCSK9 mRNA expression, densitometry of PCSK9 protein expression and representative Western blots from liver tissue. **(C)** Results from qPCR analyses on PCSK9 mRNA expression, densitometry of PCSK9 protein expression and representative Western blots from adipose tissue. All data are mean ± SEM. *p < 0.05, **p < 0.01. *n* = 14 animals per group.

In order to integrate our data into the physiological context and to get an idea of which PCSK9 quantities play a role under pathological conditions *in vivo*, we investigated changes in PCSK9 release and expression in mice with diet-induced obesity. As previously described by our group (Aurich et al., 2013), such mice show a significant increase in body mass (juvenile mice: +23%; senescent mice +33%) after administration of HFD for 16 weeks. In addition, juvenile and senescent HFD mice demonstrate a significant increase in plasma insulin, leptin and free FA (Aurich et al., 2013). The current analyses of plasma PCSK9 levels in these mice now revealed a strong increase in young HFD mice compared to animals receiving control diet or LFD. Increasing age induced a further mild increase in plasma PCSK9 in old HFD mice but a strong increase in old control and LFD mice, resulting in plasma levels comparable to young HFD mice (Figure 3A). Similarly, hepatic PCSK9 mRNA and protein expression were increased in young and old HFD mice (Figure 3B). No difference in hepatic LDL-R, LRP1 or CD36 mRNA expression was observed in these samples (Supplementary Figure 4A). Liver PCSK9 protein expression was only mildly increased in old control and old LFD mice, suggesting that other sources might play a role in the observed age-dependent increase in plasma PCSK9 in old animals (Figures 3A,B). As shown in Supplementary Figures 2C–E, visceral adipose tissue might be another important PCSK9 source *in vivo*. Therefore, we also analyzed adipose tissue PCSK9 mRNA and protein expression in those mice with diet-induced obesity. We were able to show that PCSK9 protein expression was increased in response to HFD and increasing age, but PCSK9 mRNA showed an according increase only in old LFD and HFD mice (Figure 3C). No difference in LDL-R mRNA expression was observed in response to HFD in adipose tissue, but HFD resulted in an increased expression of LRP1 and CD36 mRNA in old mice (Supplementary Figure 4B). Cardiac PCSK9 expression was largely unaffected by diet or age and remained low (not shown), similar to the data shown above for basal conditions (Supplementary Figures 2C,D, 3A). Nevertheless, circulating, exogenous PCSK9 can mediate significant effects on cardiomyocytes, which could become increasingly important in conditions with increased PCSK9 blood release such as obesity or diabetes mellitus. Therefore, we next tested the influence of exogenous PCSK9 on cardiomyocytes. For this purpose, we utilized 0.5 μg/ml of exogenous recombinant PCSK9 which should be within the upper range observed *in vivo* (Cariou et al., 2013; Girona et al., 2016).

### Impact of Exogenous PCSK9 on CTRP9-Induced Signal Transduction

**FIGURE 4:**
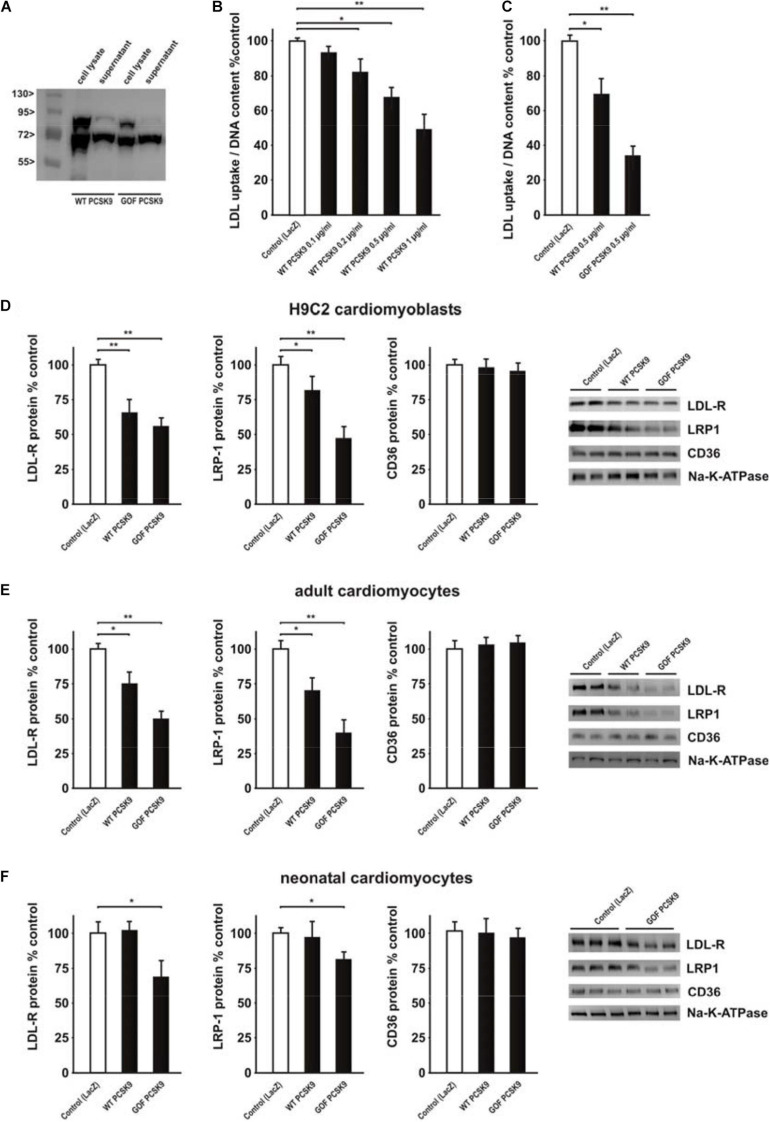
Impact of exogenous PCSK9 on LDL uptake and LDL-R or LRP1 protein expression. **(A)** HepG2 cells were treated with an adenovirus expressing murine wild-type PCSK9 or a gain-of-function (GOF) variant of PCSK9 (D377Y). Cell lysates and supernatants of HepG2 cells were collected 48 h after infection and analyzed by Western Blotting. **(B)** H9C2 cells were treated with murine wild-type PCSK9 at the indicated concentration for 24 h and LDL uptake normalized per DNA content as a measure of cell number was analyzed afterward. **(C)** H9C2 cells were treated with murine wild-type PCSK9 or GOF PCSK9 (each 0.5 μg/ml) for 24 h and LDL uptake was analyzed afterward. **(D)** H9C2 cells were treated with murine wild-type PCSK9 or GOF PCSK9 (each 0.5 μg/ml) for 24 h and afterward the membrane and cytosolic fraction were isolated from these cells. Representative Western blots for LDL-R, LRP1 or CD36 and densitometry of the according protein data are shown. Na-K-ATPase served as a loading control. **(E)** Adult rat cardiomyocytes were treated and analyzed as described in **(D)**. **(F)** Neonatal rat cardiomyocytes were treated and analyzed as described in **(D)**. All data are mean ± SEM, 4 independent experiments, *n* = 8–24 per group, *p < 0.05, **p < 0.01.

Recombinant mouse wildtype (WT) or mutant PCSK9, containing a GOF mutation (D377Y, GOF), were produced in HepG2 cells. As shown in Figure 4A, HepG2 cell lysates contained mature as well as precursor PCSK9 while the supernatant contained mainly mature PCSK9. The latter was utilized in all further experiments. In order to test the activity of PCSK9 isolated from the supernatant of HepG2 cells, we measured diI-LDL uptake in a cell-based assay as described previously (Benjannet et al., 2010). H9C2 cells demonstrated a significant decrease in LDL uptake after incubation with increasing amounts of mouse WT PCSK9 (Figure 4B). The attenuation of LDL uptake following incubation with 0.5 μg/ml GOF PCSK9 was significantly stronger compared to 0.5 μg/ml WT PCSK9, suggesting major differences in PCSK9 activity between WT PCSK9 and GOF PCSK9 (Figure 4C). Similar to the results obtained in the PCSK9 activity assay, the reduction in LDL-R and LRP1 protein expression (Figure 4D) was demonstrable in H9C2 cells following incubation with exogenous PCSK9. WT and GOF PCSK9 were similarly efficient in reducing LDL-R protein expression but the reduction of LRP-1 protein was more pronounced following treatment with GOF PCSK9 (Figure 4D). CD36, which was shown to be targeted by PCSK9 in adipocytes and hepatocytes (Demers et al., 2015), was not altered by exogenous PCSK9 in H9C2 cells (Figure 4D). Estimation of PCSK9 activity by the LDL uptake assay turned out to be highly variable in primary rat cardiomyocytes. However, Western blot analyses revealed a comparable, PCSK9-mediated reduction of LDL-R and LRP1 protein expression in adult rat cardiomyocytes (Figure 4E). GOF PCSK9 was more efficient in reducing the protein expression of both receptors in adult rat cardiomyocytes (Figure 4E). However, in neonatal rat cardiomyocytes, which show a significantly higher LRP1 and LDL-R mRNA expression than adult rat cardiomyocytes or H9C2 cells (Supplementary Figure 4C), only GOF PCSK9 was sufficient to reduce LDL-R and LRP1 protein expression (Figure 4F). CD36, which is highly expressed on the mRNA level in adult and neonatal rat cardiomyocytes compared to H9C2 cells (Supplementary Figure 4C), was not altered by exogenous PCSK9 in primary cardiomyocytes (Figures 4E,F). This suggests that exogenous PCSK9 is able to mediate similar effects on LRP1 and LDL-R protein without altering CD36 protein expression (Figures 4D–F) in H9C2 cells and in primary cardiomyocytes. All further experiments were performed with 0.5 μg/ml PCSK9, focusing largely on effects in primary cardiomyocytes.

**FIGURE 5:**
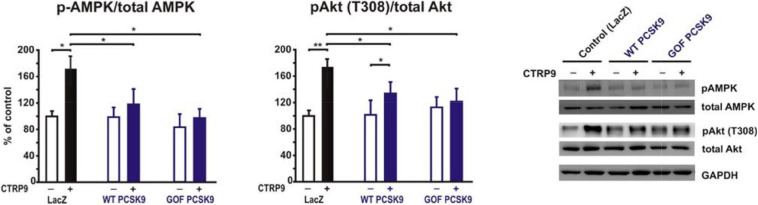
Impact of PCSK9 on CTPR9-induced signal transduction. Adult rat cardiomyocytes were incubated in the presence of LacZ-containing supernatant (control), murine wild-type PCSK9 or GOF PCSK9 (each 0.5 μg/ml) in serum-free medium for 24 h and subsequently treated with CTRP9 (4 μg/ml) for 20 min. Activation of AMPK and Akt was analyzed by Western Blotting. Representative Western blots and densitometry of protein data are shown. Total AMPK and Akt as well as GAPDH served as a loading control. All data are mean ± SEM, 4 independent experiments, *n* = 8–12 per group, *p < 0.05; **p < 0.01.

The above described impact on LDL-R and LRP1 abundance in the plasma membrane of cardiomyocytes suggests a strong potential of exogenous PCSK9 on mediating manifold effects on signal transduction and downstream cellular functions. Among the signaling pathways activated by CTRP9 are AMPK and protein kinase B (PKB), also known as Akt. Adult cardiomyocytes pre-treated for 24 h with the supernatant of LacZ expressing cells (control conditions) showed a significant increase in the activation of AMPK and Akt in response to CTRP9 (Figure 5). Most of these effects were blunted after pretreatment with PCSK9 (Figure 5). Similarly, the activation of these signaling pathways in response to adiponectin was largely abolished (Supplementary Figure 5), suggesting that LRP1 may be involved in CTRP and in adiponectin signaling.

### Impact of Exogenous PCSK9 on CTRP9-Induced Metabolic Effects

**FIGURE 6:**
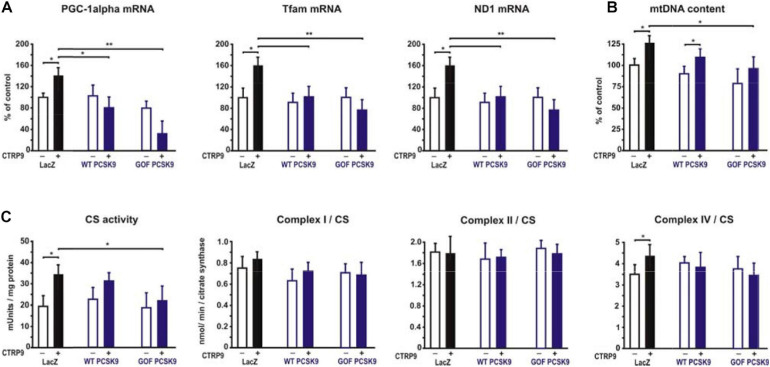
Impact of PCSK9 on CTPR9-induced mitochondrial effects. Adult rat cardiomyocytes were incubated in the presence of LacZ-containing supernatant (control), murine wild-type PCSK9 or GOF PCSK9 (each 0.5 μg/ml) in serum-free medium for 24 h and subsequently treated with CTRP9 (4 μg/ml) for 24 h. **(A)** Changes in PGC1alpha, Tfam, and ND1 mRNA expression were analyzed by Real-time PCR. **(B)** Genomic DNA was isolated from the cells and afterward analyzed by Real-time PCR for the expression of a mitochondrial transcript and a nuclear transcript in order to estimate mtDNA content. **(C)** Citrate synthase (CS) activity and the activity of complex I, complex II and complex IV of the respiratory chain were measured in cardiomyocyte lysates. All values were normalized to total protein content of the samples. All data are mean ± SEM, 4 independent experiments, *n* = 8 per group, *p < 0.05, **p < 0.01.

**FIGURE 7:**
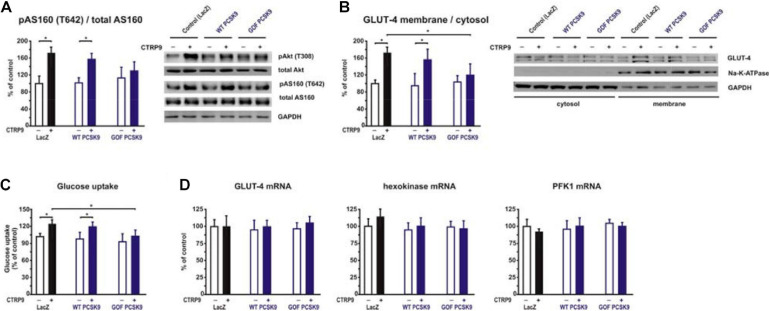
Impact of PCSK9 on CTPR9-induced effects on glucose metabolism. Adult rat cardiomyocytes were incubated in the presence of LacZ-containing supernatant (control), murine wild-type PCSK9 or GOF PCSK9 (each 0.5 μg/ml) in serum-free medium for 24 h and subsequently treated with CTRP9 (4 μg/ml) for the indicated times. **(A)** Phosphorylation of Akt and AS160 was analyzed after 20 min CTRP9 stimulation by Western Blotting. Representative Western blots and densitometry of protein data are shown. Total Akt and total AS160 as well as GAPDH served as a loading control. **(B)** Translocation of GLUT-4 from the cytosol to the plasma membrane was analyzed after 30 min CTRP9 stimulation. Representative Western blots and densitometry of protein data are shown. Na-K-ATPase served as a marker for membrane fraction and GAPDH as a marker for the cytosol. **(C)** Glucose uptake was measured after 10 min stimulation with CTRP9. **(D)** Changes in GLUT-4, hexokinase and PFK1 mRNA expression after 24 h stimulation with CTRP9 were analyzed by Real-time PCR. All data are mean ± SEM, 4 independent experiments, *n* = 8 per group, *p < 0.05.

**FIGURE 8:**
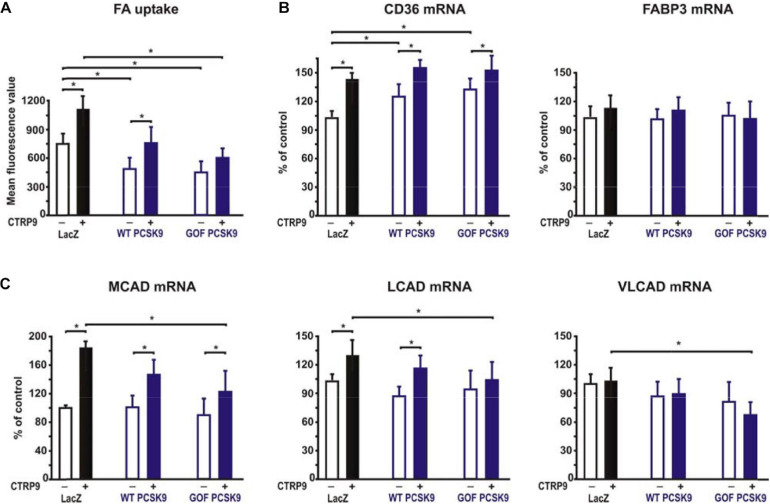
Impact of PCSK9 on CTPR9-induced effects on fatty acid metabolism. Adult rat cardiomyocytes were incubated in the presence of LacZ-containing supernatant (control), murine wild-type PCSK9 or GOF PCSK9 (each 0.5 μg/ml) in serum-free medium for 24 h and subsequently treated with CTRP9 (4 μg/ml) for the indicated times. **(A)** FA uptake was analyzed following 30 min stimulation with CTRP9 using the fluorescent long-chain FA analog BODIPY^TM^500/510 C1, C12. **(B,C)** Changes in the mRNA expression of the FA transporter CD36 or FABP3 **(B)** and enzymes of the FA oxidation MCAD, LCAD and VLCAD **(C)** were analyzed by Real-time PCR. All data are mean ± SEM, 4 independent experiments, *n* = 8 per group, *p < 0.05.

**FIGURE 9:**
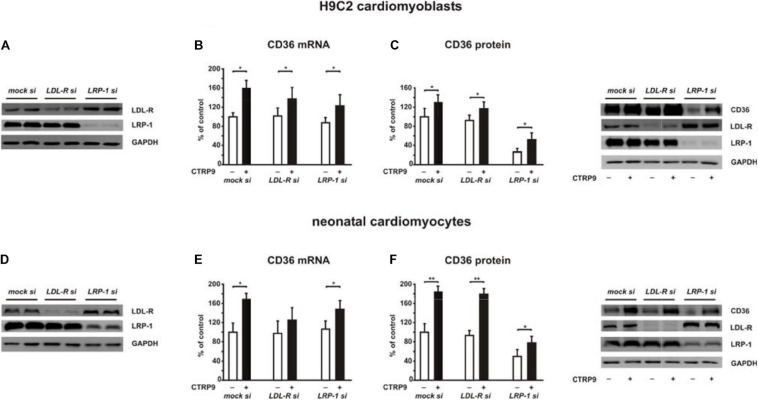
Impact of LDL-R and LRP1 on CTPR9-induced effects on fatty acid metabolism. H9C2 cells **(A–C)** or neonatal rat cardiomyocytes **(D–F)** were transfected with siRNA directed against LDL-R, LRP1 or control siRNA (mock). Cells were harvested 48 h after transfection. 24 h after transfection cells were treated with CTRP9 (4 μg/ml) as indicated. **(A,D)** Representative Western blots of LDL-R and LRP1 are shown. GAPDH served as a loading control. **(B,E)** Changes in the mRNA expression of CD36 were analyzed by Real-time PCR. **(C,F)** CD36, LDL-R, and LRP1 protein expression were analyzed by Western Blotting. Representative Western blots and densitometry of protein data are shown. GAPDH served as a loading control. All data are mean ± SEM, 4 independent experiments, *n* = 8 per group, *p < 0.05, **p < 0.01.

Adiponectin and various CTRPs mediate manifold effects on cardiomyocyte metabolism via activation of AMPK. Therefore, we next investigated whether the above described changes in signaling pathway activation also result in alterations in the metabolic effects of CTRP9 on mitochondria, glucose and FA utilization. First, we investigated markers of mitochondrial biogenesis. As shown in Figure 6, pre-treatment of adult cardiomyocytes with exogenous PCSK9 resulted in a blunted transcriptional response of the transcriptional regulators PGC-1alpha and Tfam or the primary mitochondrial transcript ND1 after CTRP9 treatment compared to the LacZ control (Figure 6A). Similarly, the increase in mtDNA content and citrate synthase activity was significantly reduced after pre-treatment with GOF PCSK9 (Figures 6B,C). However, the activity of the respiratory chain complex I and complex II were not altered in response to PCSK9 pre-treatment or CTRP9 stimulation. Complex IV on the other hand showed a significantly increased activity following CTRP9 stimulation, an effect that was blunted after PCSK9 pre-treatment (Figure 6C). As described above, PCSK9 pre-treatment also blunted the CTRP9-induced activation of Akt, a major regulator of glucose uptake via increased phospho-AS160-mediated GLUT-4 translocation. Indeed, the CTRP9-induced phosphorylation of AS160 was significantly blunted following pre-treatment with GOF PCSK9 but not with WT PCSK9 (Figure 7A). Similarly, GLUT-4 translocation and glucose uptake were increased after CTRP9 treatment in control cells or cells after treatment with WT PCSK9 while pre-treatment with GOF PCSK9 blunted these CTRP9 effects (Figures 7B,C). No changes were observed in the mRNA expression of GLUT-4, hexokinase or PFK1 in response to CTRP9 or PCSK9 pre-treatment in adult cardiomyocytes (Figure 7D). Unlike the impact on glucose metabolism, differential effects on FA metabolism were observed after PCSK9 pre-treatment in adult cardiomyocytes (Figure 8). Interestingly, WT and GOF PCSK9 reduced basal as well as CTRP9-induced FA uptake and increased basal mRNA expression of the FA translocase CD36 (Figures 8A,B). As described above, exogenous PCSK9 did not alter CD36 protein expression in adult cardiomyocytes (Figure 4E). Accordingly, no correlation was observed between CD36 protein expression and the significantly reduced FA uptake (Figure 8A) under these experimental conditions. The CTRP9-induced increase in the mRNA expression of CD36 or of the enzymes involved in FA oxidation MCAD and LCAD remained largely unaffected by PCSK9 (Figures 8B,C). In order to elucidate the impact of LRP1 and LDL-R on CTRP9-induced CD36 expression, we performed knockdown experiments in H9C2 cells and neonatal cardiomyocytes. As shown in Figures 9A,D, siRNA treatment resulted in a strong reduction of LRP1 and LDL-R protein in both cell types. The CTRP9-induced increase in CD36 mRNA and protein expression, however, remained largely unaffected by these knockdowns in H9C2 cells (Figures 9B,C) and neonatal cardiomyocytes (Figures 9E,F). Interestingly, knockdown of LRP1 appeared to mediate diverse additional effects. It resulted in a mild upregulation of LDL-R protein expression and a strong downregulation of CD36 protein expression in H9C2 cells (Figure 9C) and in neonatal cardiomyocytes (Figure 9F).

## Discussion

**FIGURE 10:**
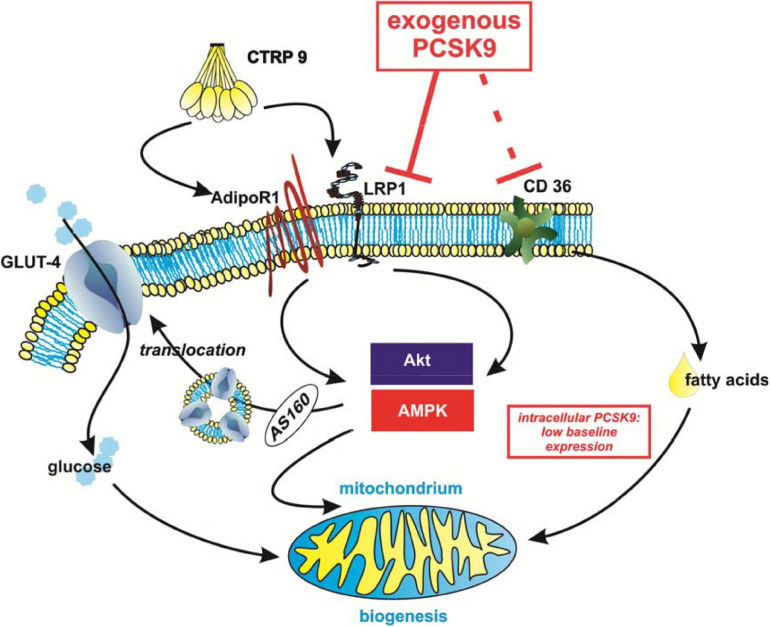
Summary of the potential impact of PCSK9 on cardiomyocyte metabolism. Although endogenous levels of PCSK9 are low in cardiomyocytes, circulating exogenous PCSK9 mediates diverse effects on metabolism via its known targets LRP1 and CD36. This has an impact on Akt- or AMPK-mediated glucose uptake and mitochondrial biogenesis in cardiomyocytes. The effects on fatty acid uptake appear to be at least in part independent from PCSK9-mediated changes in CD36 protein expression and LRP1 in cardiomyocytes.

Although the adiponectin receptors have been shown to be involved in CTRP9 signaling (Zheng et al., 2011; Kambara et al., 2012), a concomitant knockdown of both adiponectin receptors does not abolish the anti-oxidant CTRP9 effects as shown recently by our group (Niemann et al., 2020), suggesting the existence of additional CTRP9 receptors. The present study shows for the first time that LRP1 is involved in CTRP9-induced signal transduction and cellular effects in cardiomyocytes. Treatment with exogenous PCSK9 results in a significant reduction in LRP1 expression, suggesting that LRP1 is targeted by PCSK9 in cardiomyocytes. This PCSK9-mediated reduction in LRP1 subsequently diminishes CTRP9-induced signaling cascade activation, mitochondrial biogenic effects and glucose uptake in cardiomyocytes as summarized in Figure 10. Although PCSK9 also reduces basal as well as CTRP9-induced FA uptake, it does not modify CTRP9-induced changes in gene expression of FA transporters or enzymes involved in FA oxidation.

PCSK9 shows a high expression in liver, jejunum or ileum but a low expression in the heart (Seidah et al., 2003), which was confirmed by our study. Due to the low PCSK9 expression in cardiomyocytes themselves, the knockdown of endogenous PCSK9 does not lead to a change in LRP1 expression or significant alterations in cellular functions. Exogenous PCSK9, however, has a major impact on signal transduction and various metabolic functions in cardiomyocytes via LRP1. The effects of GOF PCSK9 (Lu et al., 2016), which results in sustained hypercholesterolemia and increased atherosclerotic lesions in mice *in vivo*, are consistently more pronounced than the effects of WT PCSK9 in our study. Previously, oxLDL, hypoxia or TNF-alpha have been shown to induce the expression of PCSK9 in adult rat cardiomyocytes or neonatal mouse cardiomyocytes (Schluter et al., 2017; Ding et al., 2018), and a positive effect of PCSK9 inhibition on cardiac function after ischemia or ischemia/reperfusion injury has been demonstrated *in vivo* in mice and rats (Ding et al., 2018; Palee et al., 2019). However, only when PCSK9 expression is increased by oxLDL, the threshold of PCSK9 is sufficient to negatively affect contractile function of cardiomyocytes (Schluter et al., 2017). Therefore, we cannot rule out functional metabolic consequences in cardiomyocytes in situations with an increased endogenous PCSK9 expression.

Various studies have found increased plasma PCSK9 levels in patients suffering from type 2 diabetes mellitus, metabolic syndrome or obesity (Girona et al., 2016; Levenson et al., 2017; Dijk and Cariou, 2019). Furthermore, even short-term application of hypercaloric diets results in increased plasma PCSK9 in young healthy volunteers, reaching PCSK9 plasma levels of up to 500 ng/ml (Cariou et al., 2013), the concentration of exogenous PCSK9 in our *in vitro* experiments. Similarly, our analyses in mice with diet-induced obesity demonstrate an increase of PCSK9 plasma levels from 100 ng/ml (control animals) to up to 350 ng/ml (HFD animals). The previously observed increase in plasma insulin levels in the young HFD and old control, LFD or HFD mice (Aurich et al., 2013) may have contributed to the increased hepatic PCSK9 expression and plasma PCSK9 release via SREBP-1c as suggested by others in rodents (Costet et al., 2006; Krysa et al., 2017). In addition, the lipid composition of the HFD plays a major role in the regulation of PCSK9 plasma concentrations, because cholesterol, saturated FA and polyunsaturated FA mediate diverging effects on PCSK9 expression as summarized by others (Krysa et al., 2017). Recently, it has been shown that hepatic steatosis following HFD results in the induction of liver PCSK9 mRNA and protein, a process involving saturated FA-induced, SREBP2-driven *de novo* PCSK9 expression (Lebeau et al., 2019b). There is an ongoing controversy regarding the expression and release of PCSK9 from adipose tissue. While Roubtsova et al. (2011) reported very low levels of PCSK9 mRNA in mouse perigonadal fat, others have shown that PCSK9 is abundantly expressed in human visceral and epicardial adipose tissue and secreted by cultured, mature adipocytes (Bordicchia et al., 2019; Dozio et al., 2020) and that a positive correlation with the body mass index in humans exists (Bordicchia et al., 2019). Our data suggest a similar effect in mice with diet-induced obesity: While juvenile mice showed a strong increase of liver and fat PCSK9 protein expression, at higher age adipose tissue might contribute to plasma PCSK9. Hepatic PCSK9 is the major source of circulating PCSK9 and thus most likely responsible for its systemic effects. Nevertheless, our study confirms that adipose tissue expresses and secretes PCSK9, although the amount is significantly lower than in hepatic tissue (Supplementary Figures 2C–E). Accordingly, local effects of epicardium-derived PCSK9 (Dozio et al., 2020) can be envisioned in the human heart due to the lack of a barrier between epicardial adipose tissue and the myocardium and due to their shared microcirculation. Such an interaction has been demonstrated for adipocytokines previously (Venteclef et al., 2013). Whether the amount of PCSK9 secreted from epicardial adipose tissue is sufficient to modify myocardial LRP1 protein levels requires further investigations.

Although FA are the predominant substrate used by the adult heart, healthy cardiomyocytes are metabolically versatile in using other available substrates such as glucose, lactate, amino acids, and ketone bodies (Niemann et al., 2018). This high level of metabolic flexibility helps to ensure energy supply in response to stressful conditions. However, heart failure is characterized by mitochondrial dysfunction and a loss of metabolic flexibility (Niemann et al., 2018). The results of our study suggest that PCSK9 inhibition in patients is likely to exert beneficial cardiac effects beyond lipid lowering at least in part via AMPK-mediated mitochondrial biogenesis. Similarly, PCSK9 inhibition in a rat model of diet-induced obesity or prior to ischemia was shown to mediate cardioprotection through preservation of cardiac mitochondrial function, reduction in mitochondrial reactive oxygen species and attenuation of mitochondrial apoptosis leading to improved LV function (Palee et al., 2019; Amput et al., 2020).

In addition to AMPK, CTRP9 also activates PKB/Akt. This protein kinase affects glucose metabolism in different ways including increased translocation of the glucose transporter GLUT-4 to the plasma membrane, increased expression of GLUT-1 and hexokinase or phosphorylation of GSK3 (Nicholson and Anderson, 2002). In particular the phosphorylation of AS160, a Rab GTPase-activating protein involved in the rapid and reversible redistribution of GLUT-4 from intracellular vesicles to the plasma membrane, is involved in the increased influx of glucose into cells (Mafakheri et al., 2018). We observed an attenuation of the CTRP9-induced AS160 phosphorylation at Thr642, a phosphorylation site for both PKB/Akt and AMPK (Mafakheri et al., 2018), following GOF PCSK9 but not WT PCSK9 treatment. The reduced AS160 phosphorylation results in a decline in GLUT-4 translocation to the membrane and a reduced glucose uptake in response to CTRP9 in adult rat cardiomyocytes, suggesting that exogenous PCSK9 impairs glucose metabolism. Whether these effects depend on additional signaling steps upstream from AS160 and in addition to AMPK needs to be investigated in future studies. Altogether these data imply that PCSK9 inhibition may mediate cardioprotective effects also via glucose metabolism. This is an important finding, as both data from PCSK9 KO mice and data from patients with genetic PCSK9 variants indicated an increased risk of type 2 diabetes mellitus (Mbikay et al., 2010; Schmidt et al., 2017). Whereas enhancing the LDL-R pathway in the liver is beneficial and antiatherogenic, activating the same pathway in pancreatic beta cells may lead to a harmful cholesterol overload, increased cell death of beta cells and development of diabetes (Roehrich et al., 2003). However, the currently available data from patients with anti-PCSK9 therapies do not show an increased incidence of diabetes (Sabatine et al., 2017).

The observed reduction of FA uptake is in accordance with reports showing that PCSK9 induces the degradation of CD36 via a proteasome-sensitive mechanism, resulting in reduced long-chain FA uptake in adipocytes and hepatocytes (Demers et al., 2015; Lebeau et al., 2019a). In addition, loss of PCSK9 has also been shown to result in hepatic injury in mice with diet-induced obesity, an effect largely attributed to the effects of PCSK9 on its target CD36 (Lebeau et al., 2019a). However, we did not detect significant changes in CD36 protein expression in any of the cardiac cells *in vitro*, suggesting that cell type-specific differences appear to exist in response to PCSK9 treatment. This may imply that the observed effects of PCSK9 on FA uptake are independent from LRP1-mediated mechanisms in cardiomyocytes. Similarly, LRP1 and LDL-R are not involved in the CTRP9 effects on CD36 expression in cardiomyocytes or cardiomyoblasts. Phosphorylated AS160, which is reduced following PCSK9 treatment, could represent a potential candidate involved in mediating the effects of PCSK9 on FA uptake. Indeed, AS160 mediates translocation of CD36 in cardiomyocytes and was suggested to function as a hub that integrates regulation of GLUT-4 and CD36 translocation in cardiomyocytes (Samovski et al., 2012). The reduced AS160 phosphorylation at Thr642 may thus not only contribute to the reduced GLUT-4 translocation but also to the reduced FA uptake despite unchanged CD36 protein expression. Although knockdown of LRP1 had no impact on the effects of CTRP9 on CD36 protein expression, it resulted in a strong reduction of basal CD36 protein expression (Figure 9) and a reduction of fatty acid uptake (not shown) compared to mock transfected cells. Therefore, future investigations need to elucidate the impact of LRP1 on basal CD36 protein expression together with the role of PCSK9 in this process.

The heart is a complex organ consisting of a variety of different cells among those are cardiomyocytes, endothelial cells, fibroblasts, vascular smooth muscle cells, pacemaker cells and immune cells. *In vivo* studies are necessary in order to investigate the interactions between these different cell types. In addition, there is a large demand for *in vitro* cardiac-like cell models, and the reductionist approach inherent to all cell cultures is inevitable to analyze signal transduction, cell behavior and responses to pharmaceuticals or toxic substances of individual cells. However, currently there is no ideal *in vitro* model that mimics all properties of cardiomyocytes *in vivo*. Adult cardiomyocytes are fragile cells that can be kept in culture without de-differentiation only for a limited time. Therefore, we also utilized primary neonatal rat cardiomyocytes and H9C2 cardiomyoblasts in our study. The H9C2 myoblast cell line, isolated from rat ventricular tissue, is widely used *in vitro* as a mimetic for skeletal and cardiac muscle due its biochemical, morphological, electrical or energetic properties (Hescheler et al., 1991; Kuznetsov et al., 2015). They are of major interest in cardiovascular research as they are easy to cultivate and manipulate. Our present analyses suggest that expressional profiles of PCSK9, LRP1, and LDL-R are similar to each other in adult rat cardiomyocytes and in H9C2 cells, making them a suitable model for the analysis of PCSK9-related signal transduction.

In summary, our study shows that PCSK9 treatment has an impact on cardiomyocyte metabolism, in part through LRP1-mediated signaling pathways in cardiomyocytes (Figure 10). Therapeutic PCSK9 inhibition may therefore not only represent a treatment of hypercholesterolemia and reduce major atherosclerotic events but also provide additional benefits beyond cholesterol metabolism through stimulation of glucose uptake and mitochondrial biogenesis thereby influencing cardiac function.

## Data Availability Statement

The raw data supporting the conclusions of this article will be made available by the authors, without undue reservation.

## Ethics Statement

The animal study was reviewed and approved by Institutional animal care and use committee of the Martin Luther University Halle-Wittenberg; Halle, Germany as described in the following manuscript: doi: 10.1007/s00395-013-0369-6.

## Author Contributions

SR designed and planned the study, performed the experiments, and wrote the manuscript. LL, TN, FK, and NM performed the experiments. BN designed and performed the experiments and analyzed the data. RS planned and wrote the manuscript. All authors contributed to the article and approved the submitted version.

## Conflict of Interest

The authors declare that the research was conducted in the absence of any commercial or financial relationships that could be construed as a potential conflict of interest.

## Abbreviations

AdipoR1: adiponectin receptor 1
Akt: protein kinas B
AMPK: AMP-dependent protein kinase
CD36: cluster of differentiation 36 = fatty acid translocase
Cox I: cytochrome oxidase I
CS: citrate synthase
CTRP9: C1q/TNF-related protein 9
FABP3: fatty acid binding protein 3
GAPDH: glyceraldehyde-3-phosphate dehydrogenase
GLUT: glucose transporter
GOF: gain of function
HFD: high fat diet
HPRT1: hypoxanthine phosphoribosyl transferase 1
LCAD: long-chain acyl-CoA dehydrogenase
LDL-R: LDL receptor
LFD: low fat diet
LRP1: Low density lipoprotein receptor-related protein 1
MCAD: medium-chain acyl-CoA dehydrogenase
mtDNA: mitochondrial DNA
ND1: NADH-ubiquinone oxidoreductase chain 1
oxLDL: oxidized low-density lipoprotein cholesterol
PFK1: phosphofructokinase-1
PGC-1alpha: peroxisome proliferator-activated receptor-gamma coactivator-1alpha
PCSK9: proprotein convertase subtilisin/kexin-9
VLCAD: very long-chain acyl-CoA dehydrogenase.
