# Supplementary material for: Impact of PCSK9 on CTRP9-Induced Metabolic Effects in Adult Rat Cardiomyocytes
Source: Front Physiol. 2021 Feb 11;12:593862. doi: 10.3389/fphys.2021.593862 (PMC7904879; doi:10.3389/fphys.2021.593862)
Supplement: Supplementary file 1 [file Data_Sheet_1.pdf]

Supplement to

Rohrbach et al.: Impact of PCSK9 on CTRP9-induced metabolic effects in adult rat cardiomyocytes

1. Supplementary Figures:

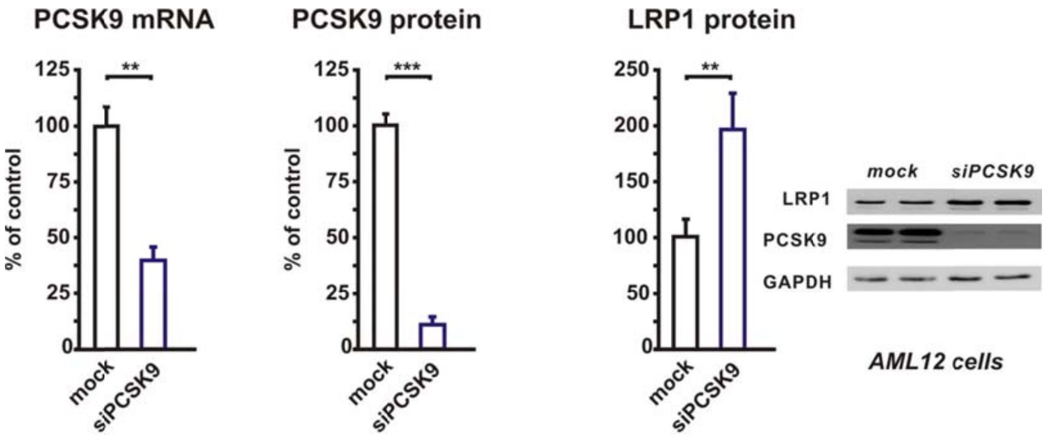

Supplementary Figure 1: Impact of PCSK9 knockdown on LRP1 expression

Mouse AML12 cells were transfected with PCSK9 siRNA or mock siRNA. Changes in PCSK9 mRNA or protein expression were analyzed by Real-time PCR or Western Blotting 48 hours after transfection. The impact of PCSK9 knockdown on LRP1 protein expression was analyzed by Western Blotting. GAPDH served as a loading control. All data are mean±SEM, 4 independent experiments, n=8-12 per group, **: $p<0.01$, ***:$p<0.001$.

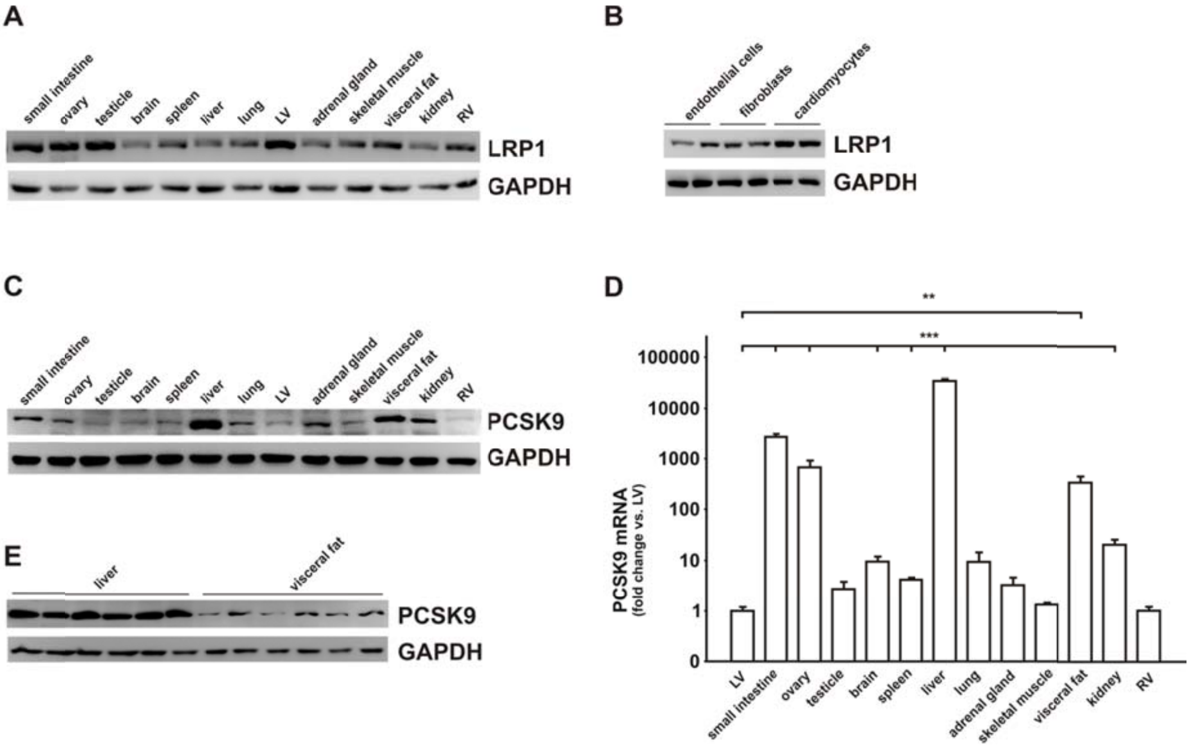

Supplementary Figure 2: Expressional patterns of LRP1 and PCSK9 protein

A: LRP1 protein expression was analyzed in various rat organs by Western Blotting. **B:** Endothelial cells, fibroblasts and cardiomyocytes were isolated from rat heart and utilized for the analysis of LRP1 protein expression by Western Blotting. **C:** PCSK9 protein expression was analyzed in various rat organs by Western Blotting. **D:** PCSK9 mRNA expression was analyzed in various rat organs by qPCR. All data are presented in comparison to LV expression. Data are mean±SEM, 4 independent experiments, n=4 per group. **: $p<0.01$; ***: $p<0.001$. **E:** PCSK9 protein expression was analyzed in rat liver tissue and visceral adipose tissue from 6 young, male rats by Western Blotting. GAPDH served as a loading control in all Western Blot analyses.

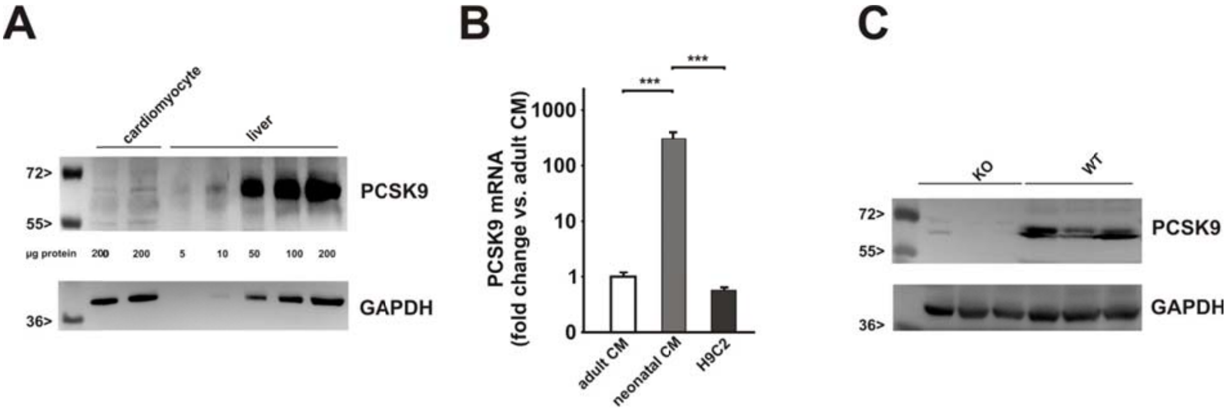

Supplementary Figure 3: PCSK9 expression in cardiomyocytes

A: PCSK9 protein expression was compared in lysates from adult rat cardiomyocytes (200 µg) and from rat liver (5-200 µg each) by Western Blotting. GAPDH served as a loading control. **B:** PCSK9 mRNA expression was analyzed in adult rat cardiomyocytes, H9C2 cells and neonatal rat cardiomyocytes by qPCR. Data are presented in comparison to the expression in adult rat cardiomyocytes. Data are mean±SEM, 3 independent experiments, n=12 per group. ***: $p<0.001$. **C:** In order to rule out unspecific binding, we performed PCSK9 antibody testing in liver tissue (25µg protein lysate) from PCSK9 knockout mice and wild-type (WT) mice [1].

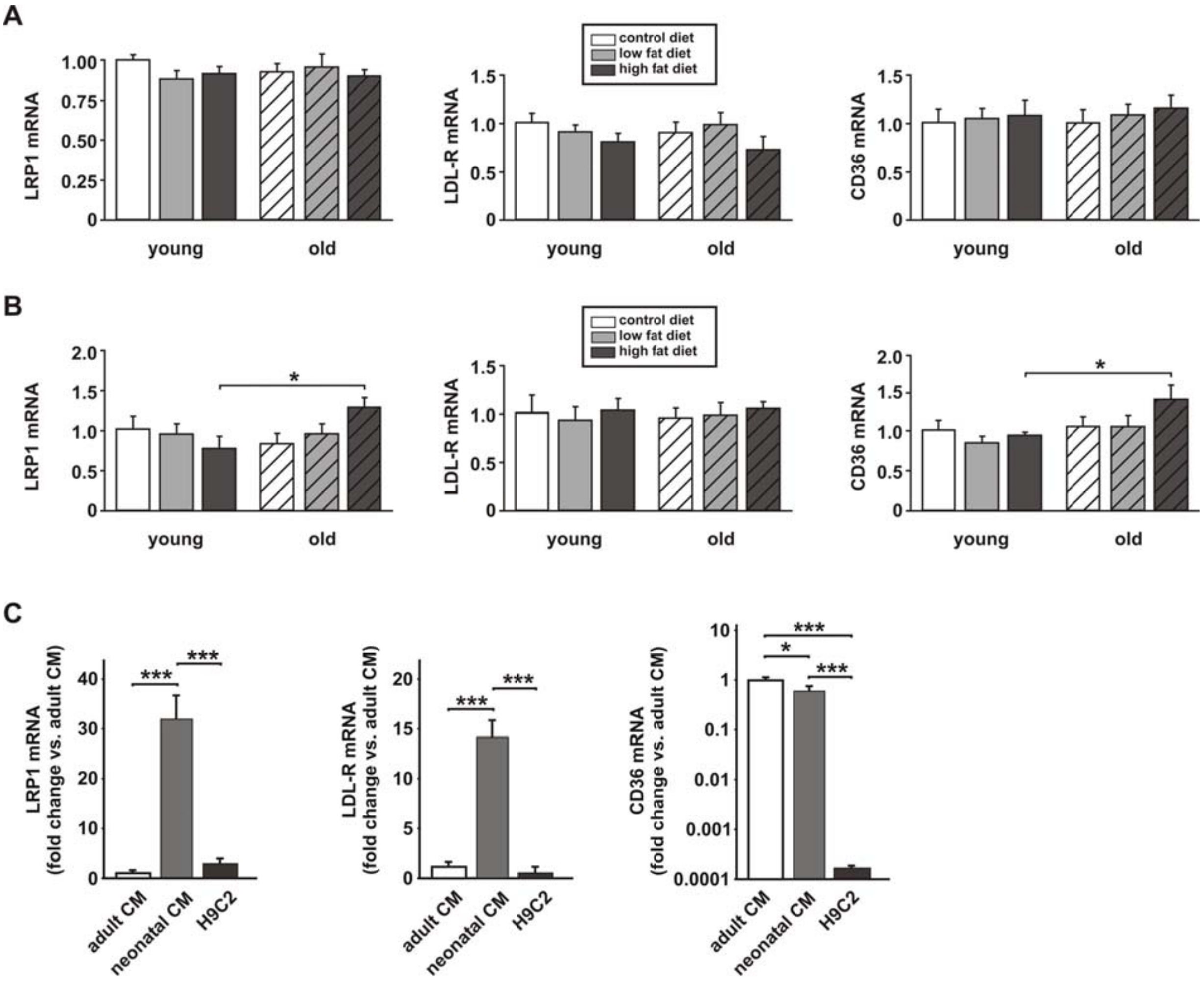

Supplementary Figure 4: LRP1, LDL-R and CD36 mRNA expression in mouse tissues and in rat cardiac cells

A: Results from qPCR analyses on LRP1, LDL-R and CD36 mRNA expression in liver tissue obtained from young and old mice receiving control diet, low fat diet (LFD) or high fat diet (HFD) for 16 weeks. **B:** Results from qPCR analyses on LRP1, LDL-R and CD36 mRNA expression in adipose tissue as described in **A**. All data are mean±SEM. n=14 animals per group. *: $p<0.05$. **C:** LRP1, LDL-R and CD36 mRNA expression were analyzed in adult rat cardiomyocytes, H9C2 cells and neonatal rat cardiomyocytes by qPCR. Data are presented in comparison to the expression in adult rat cardiomyocytes. Data are mean±SEM, 3 independent experiments, n=12 per group. *: $p<0.05$; ***: $p<0.001$.

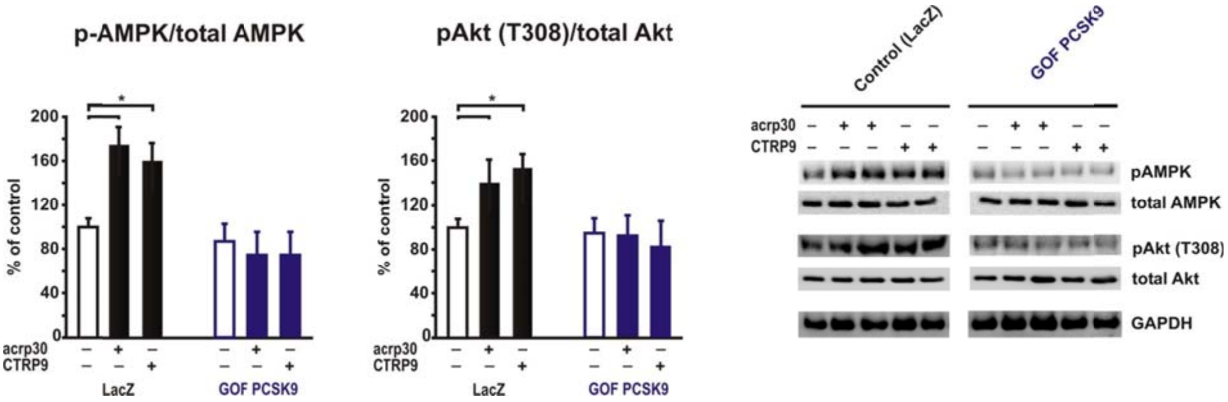

Supplementary Figure 5: Comparison of the impact of PCSK9 on adiponectin- or CTPR9-induced signal transduction

Adult rat cardiomyocytes were incubated in the presence of PCSK9 (0.5µg/ml) in serum-free medium for 24 h and treated thereafter with adiponectin (acrp30, 4µg/ml) or CTRP9 (4µg/ml) for 20 min. Activation of AMPK and Akt was analyzed by Western Blotting. Total AMPK and Akt as well as GAPDH served as a loading control. All data are mean±SEM, 4 independent experiments, n=8-12 per group, *: $p<0.05$.

2. Reference:

1. Schluter KD, Wolf A, Weber M, Schreckenberg R, Schulz R. Oxidized low-density lipoprotein (oxLDL) affects load-free cell shortening of cardiomyocytes in a proprotein convertase subtilisin/kexin 9 (PCSK9)-dependent way. Basic Res Cardiol. 2017;112:63. doi: 10.1007/s00395-017-0650-1
